# A Re-do Repair of Tetralogy of Fallot With an Anomalous Coronary Artery Using a Valved Conduit for the Right Ventricular Outflow Tract Reconstruction

**DOI:** 10.7759/cureus.61794

**Published:** 2024-06-06

**Authors:** Yoshihiro Honda, Shoji Suzuki, Shigeaki Kaga, Hiroyuki Nakajima

**Affiliations:** 1 Department of Surgery, University of Yamanashi, Yamanashi, JPN; 2 Department of Medical Education, University of Yamanashi, Yamanashi, JPN

**Keywords:** right ventricular outflow tract, anomalous coronary arteries, unplanned reoperation, pulmonary stenosis, surgery, tetralogy of fallot

## Abstract

Several techniques can be used for the repair of right ventricular outflow tract (RVOT) stenosis in patients with an anomalous coronary artery. Here, we report a case of conduit obstruction after re-operation following double-tract reconstruction and release of stenosis by main pulmonary artery transection and conduit replacement. The patient is a female child diagnosed with tetralogy of Fallot with an anomalous coronary artery (right coronary across right ventricle outflow) who underwent correction with a double-tract RVOT repair at the age of 10 months (weight: 8 kg). At the age of eight years (weight: 24 kg), a conduit re-implantation procedure was required because of an increase in body weight. Designing smooth conduits proved challenging due to the anomalous coronary artery and a short main pulmonary arterial trunk. RVOT stenosis was documented early postoperatively, and further intervention was deemed necessary. At 13 years of age (weight: 45 kg), the patient underwent implantation of an adult-size valved conduit. Transection of the main pulmonary artery and extensive mobilization of the bilateral pulmonary arteries were effective in creating a relative laminar blood flow. Postoperative evaluations confirmed that the conduit was well-shaped and had satisfactory valve functionality. This case highlights the potential difficulties involved with replacing an additional conduit after double-tract cardiac repair procedures, particularly due to anatomical constraints posed by a coronary artery and a short pulmonary arterial trunk. Main pulmonary artery transection and comprehensive mobilization of branch pulmonary arteries can be a solution to conduit design difficulties in RVOT reconstruction after double-tract cardiac repair procedures.

## Introduction

Since the advent of surgery for tetralogy of Fallot (TOF) nearly seven decades ago, primary repair of TOF has been one of the most commonly performed procedures with acceptable early and late results. A successful repair entails effective relief of right ventricular outflow tract (RVOT) obstruction. This is achieved in most cases by a right ventriculotomy followed by patch augmentation of the RVOT.

However, RVOT repair is often challenging in patients with a crossing anomalous coronary artery. Many different surgical techniques can be used for a complete repair of TOF with coronary arteries crossing the anterior wall of the right ventricle (RV). Transatrial-transpulmonary correction remains the most common technique used if possible. However, it can be avoided in the majority of patients by placing an extracardiac conduit, which has shown good clinical outcomes, and by using alternative surgical approaches [1].

Here, we report a case of RVOT obstruction after re-implantation of an additional tract for the double-tract repair of TOF with an anomalous coronary artery. The patient underwent RVOT occlusion release via transection of the main pulmonary arterial (PA) trunk and placement of a valved conduit from the RV to the PA.

## Case presentation

**Figure 1:**
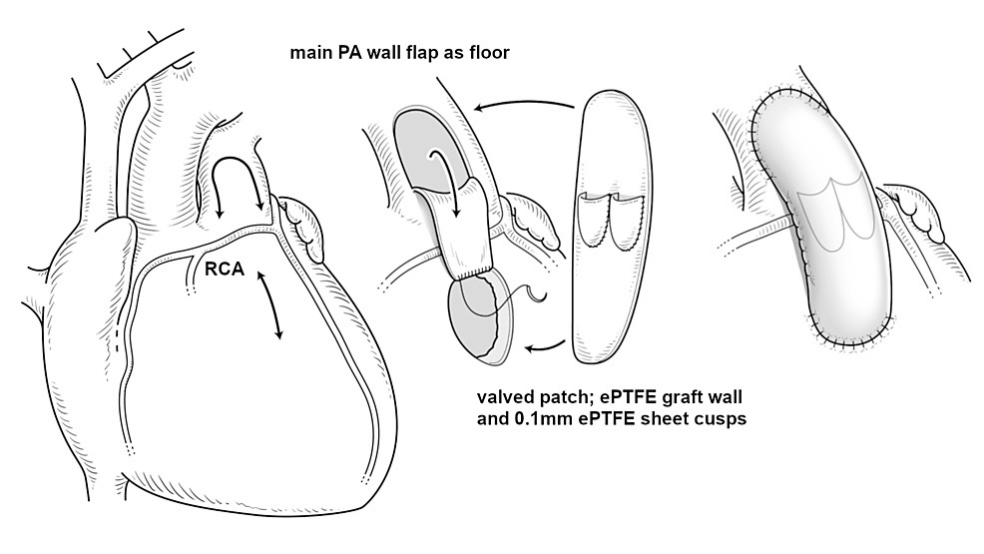
Schema of double-tract right ventricular outflow tract repair at correction An additional tract was created with a pulmonary arterial wall flap as a floor and an expanded polytetrafluoroethylene patch with two cusps as an anterior wall. RCA: right coronary artery (anomalous); PA: pulmonary artery; ePTFE: expanded polytetrafluoroethylene The image was created by Yoshihiko Tsuda of the Da Vinci Medical Illustration Office.

The patient is a female child diagnosed with TOF with an anomalous right coronary artery originating from the left anterior descending artery that crossed the RVOT in infancy. A modified Blalock-Taussig shunt with an expanded polytetrafluoroethylene (ePTFE) graft was performed at 10 months of age as an initial palliative treatment. Subsequently, the patient underwent correction of the TOF at the age of two years and eight months (weight: 10.0 kg). RVOT repair was performed using the double-tract method described by van Son [2] with a PA wall flap floor and ePTFE patch with two cusps included in the anterior wall of the repair (Figure 1). The ePTFE patch was created from a synthetic graft wall with two semilunar cusps of 0.1 mm sheet, 12 mm in width. The PA anterior wall was incised in the inverted U shape (5 mm width) and sewn to the top of the RV incision as the floor of the additional tract. The original RVOT muscle was not resected or incised annularly to minimize valve regurgitation. The created valved patch was then sewn as the anterior wall, and the ventricular septal defect was closed using the ePTFE patch.

The postoperative course was uneventful. Subsequently, the initial RVOT repair was assessed because of normal weight gain when the patient was eight years old. The original PA valve diameter was 8 mm, and no significant enlargement was observed. Owing to concerns about coronary arterial compression due to the use of an oversized graft, we opted to use a 16 mm valved conduit for replacement and to retain the native RVOT during this re-reconstruction. A valved ePTFE conduit, fabricated at the Kyoto Prefectural University of Medicine with three bulging sinuses of diameter 16 mm, was used. Due to the tight flexure of the conduit route over the anomalous coronary artery, it was difficult to determine the final length and shape of the conduit without deforming the valve. Intraoperative transesophageal echocardiogram revealed that the blood flow velocity was 2.0 m/s; RV pressure was not measured during the operation. We concluded that the shape of the new conduit was a suitable modification of the initial RVOT procedure.

**Figure 2:**
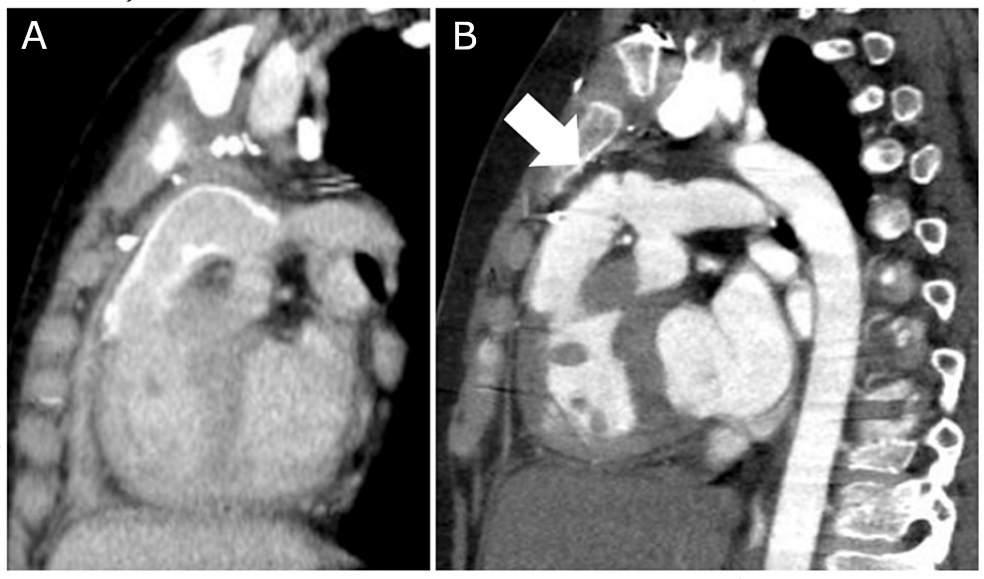
Sagittal section of computed tomography: (A) 10 days and (B) five years after re-operation. An additional tract is curved tightly, bent at the valve just above the coronary artery (white arrow) after five years.

However, follow-up echocardiography revealed a gradual increase in postoperative RV pressure. Catheter examination and computed tomography (CT), three years after this re-operation, revealed that the RV pressure had increased to 90% of that in the left ventricle; there was tight bending of the conduit over the right coronary artery, and moderate conduit valve regurgitation was present (Figure 2).

Periodic echocardiography was continued for careful follow-up of the patient. The RV pressure remained at 90% of that in the left ventricle, and tricuspid annular plane systolic excursion (TAPSE) of 8.6 mm was present with an RV fraction area change (FAC) of 0.40, indicating reduced RV function; however, no chest symptoms or signs of arrhythmia were observed. Re-RVOT reconstruction was planned when the patient was 13 years old and weighed 45.0 kg as this body weight was considered sufficient for adult-size conduit implantation without concerns for further RV dysfunction at high pressure.

**Figure 3:**
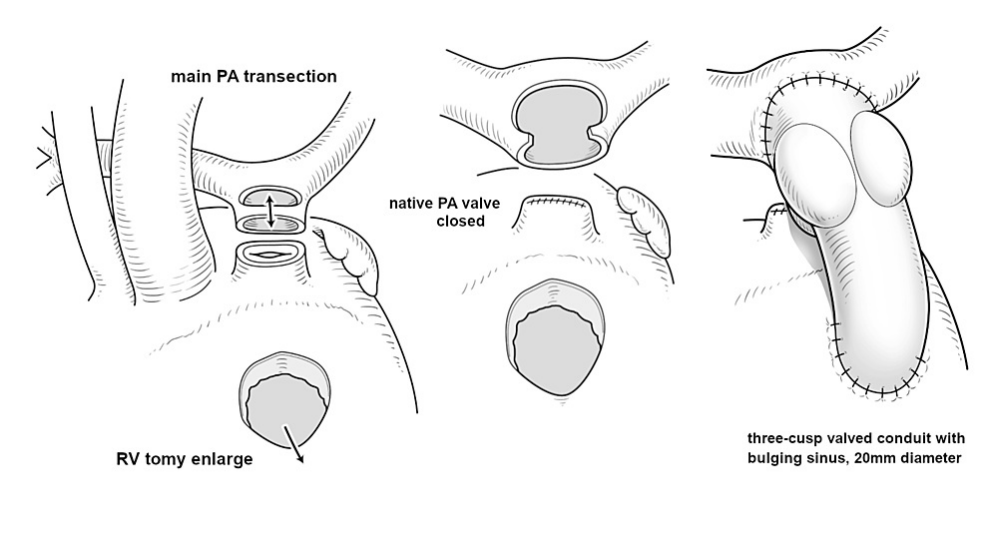
Schema of re-reoperation The main pulmonary trunk was transected, and the proximal stump was closed. A 20 mm diameter three-cusp valved conduit was implanted. RV: right ventricle; PA pulmonary artery The image was created by Yoshihiko Tsuda of the Da Vinci Medical Illustration Office.

Under the third median thoracotomy, cardiopulmonary bypass with mild hypothermia was established. The aorta was cross-clamped, and cardioplegia was introduced. The main PA trunk was transected, and the proximal stump of the PA was sutured and closed. The bilateral PA branches were dissected and mobilized to the bilateral hilum of the lung. A crafted 20 mm diameter three-cusp valved ePTFE conduit was implanted. The conduit valve was positioned beneath the PA bifurcation to avoid valve deformation (Figure 3).

**Figure 4:**
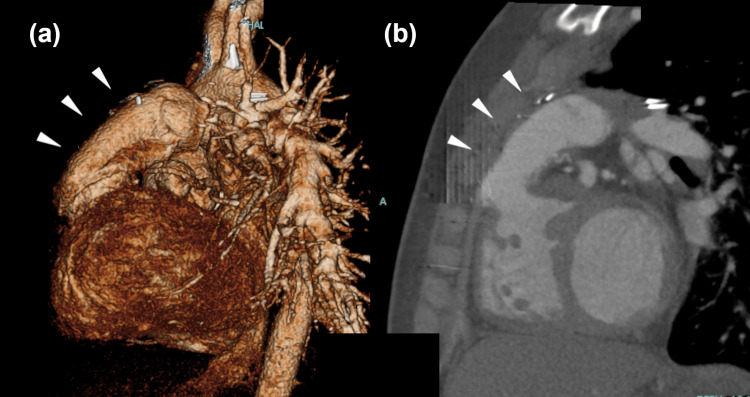
Computed tomography images three months after re-reoperation: (a) Volume rendered image, left side view; (b) similar sagittal image. Note the smooth and severe flexion-free form of conduit (arrows).

The patient’s postoperative course was uneventful with no ischemic heart events or arrhythmia. An echocardiogram obtained eight days postoperatively and CT images obtained three months postoperatively revealed decreased RV pressure and no regurgitation or bending of the conduit (Figure 4).

**Figure 5:**
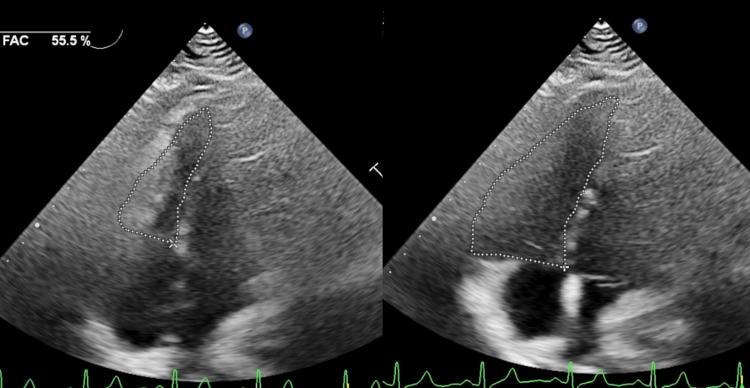
Echocardiography of right ventricular function five years after re-reoperation FAC: fractional area change

One year postoperatively, a catheter examination revealed the absence of RVOT regurgitation and decreased RV systolic end-diastolic pressure (30/9 mmHg). Echocardiographic evaluation of RV function showed that the TAPSE was 15.2 mm and FAC was 44.3%, and improved to 55.5% at five years after re-reoperation (Figure 5). The patient has maintained New York Heart Association class I activity six years after conduit replacement and continues to undergo periodic echocardiography-based evaluations.

## Discussion

The present case highlights two important points. First, replacing an additional conduit after a double-tract repair can be challenging due to the narrow, curved route limited by an underlying coronary artery and a short main pulmonary trunk. Second, main PA transection and mobilization of the bilateral PAs through wide dissection facilitates a more optimal conduit shape, which enhances the efficacy of outflow reconstruction after double-tract surgical repair.

The prevalence of coronary arterial anomalies in TOF is reported as 4-6%, and a surgical approach should be adopted for RVOT repair [3]. Several techniques - such as trans-atrial approach with or without pulmonary arteriotomy, double tract operation with a PA flap and patch, two-patch augmentation with a curved transannular oblique patch and an infundibular patch, oblique ventriculotomy with a subcoronary suture line, main PA transection and translocation to RV incision, and transannular patch with coronary artery-wide mobilization - have been reported for RVOT repair in patients with TOF with an anomalous coronary artery [1,4-7]. Although double-tract repair is associated with good short-term outcomes [6], there are very few reports regarding long-term outcomes. Notably, growth of the native RVOT has been reported during the follow-up period after double-tract repair [5,7]; however, significant native RVOT growth was not observed in the present case. Although we did not perform RVOT myocardial resection to minimize pulmonary regurgitation, reported cases emphasize the importance of myocardial resection of the trans-atrial and trans-pulmonary arteries, which can be important with regard to the potential for RVOT growth and may reduce the possibility of an increase in RVOT pressure.

The additional tract created in this current operative case was well maintained without occlusion until the patient obtained a body weight of 24 kg. However, at the time of conduit re-implantation, ensuring that the conduit was smoothly shaped was difficult, as the bending route was tightly constrained due to the short main PA trunk and underlying coronary artery. The main PA trunk transection and wide dissection to the bilateral hilum were useful for achieving sufficient mobilization of the PA to create a smooth route without coronary artery compression. The use of a fabricated valved ePTFE conduit with fan-shaped leaflets and a bulging sinus has been reported to exhibit excellent mid- to long-term valve function and durability [8]. It must be noted that RVOT reconstruction using a conduit is considered a risk factor for remote restenosis and reoperation owing to the lack of growth potential [1,7,9]; however, replacement with a conduit appropriate for adults can reduce the risk of restenosis.

In this case, valve deformation caused by conduit bending could also have impaired valve function. Alternatively, the use of a conduit with a stented bioprosthetic valve may be effective; however, size limitations (minimum size of 19 mm) and long-term durability will need to be considered. Transcatheter valve implantation is an option in case of further degeneration of bioprosthetic valves [10,11].

## Conclusions

Double-tract repair is one of the surgical techniques used for RVOT reconstruction in cases of an anomalous coronary artery. From a plethora of available techniques, the one that works best in a particular situation needs to be chosen depending on the surgeon's preference. Repair using a conduit should be the last resort.

This case illustrated the potential difficulties with repairing the additional tract created during double-tract repair, as well as the effectiveness of the main PA transection, mobilization, and replacement with an adult-size conduit. Thus, the RVOT reconstruction method should be considered along with further reintervention strategies after long-term follow-up.
